# Development and evaluation of training resources to prepare health professionals for counselling pregnant women about non-invasive prenatal testing for Down syndrome: a mixed methods study

**DOI:** 10.1186/s12884-017-1315-7

**Published:** 2017-04-27

**Authors:** Kerry Oxenford, Rebecca Daley, Celine Lewis, Melissa Hill, Lyn S. Chitty

**Affiliations:** 1 Fetal Medicine Unit, University College London Hospitals NHS Foundation Trust, London, UK; 2 NE Thames Regional Genetics Service, Great Ormond Street Hospital for Children NHS Foundation Trust, London, UK; 3 Genetics and Genomic Medicine, UCL Great Ormond Street Institute of Child Health, London, UK

**Keywords:** Non-invasive prenatal testing, Down syndrome, Training, Education, Health professionals, Midwives

## Abstract

**Background:**

The availability of non-invasive prenatal testing (NIPT) for aneuploidies is expanding rapidly throughout the world. Training health professionals to offer NIPT in a way that supports informed choice is essential for implementation. The aim of this study was to develop and evaluate a training package for health professionals to support the introduction of NIPT into clinical practice.

**Methods:**

Training on NIPT was offered to health professionals, primarily midwives, involved in Down syndrome screening and testing in eight hospitals located in England and Scotland as part of a research study evaluating the implementation of NIPT in the UK National Health Service. Training was evaluated using a mixed methods approach that included quantitative questionnaires at three time points and post-training qualitative interviews. The questionnaires measured confidence, self-perceived knowledge and actual knowledge about NIPT for Down syndrome. Interviews explored opinions about the training and experiences of offering NIPT.

**Results:**

The training provided to the health professionals was found to positively impact on their confidence in discussing NIPT with women in their clinic, and both their perceived and actual knowledge and understanding of NIPT was improved. Knowledge remained weak in four areas; cell-free fetal DNA levels increase with gestation; turnaround time for NIPT results; cell-free fetal DNA is placental in origin; and NIPT false positive rate.

**Conclusions:**

Training materials, including a lesson plan, PowerPoint presentation and written factsheet on NIPT, have been developed and evaluated for use in educating midwives and supporting the introduction of NIPT. Implementation of training should include a greater focus on the areas where knowledge remained low. Some groups of midwives will need additional training or support to optimise their confidence in discussing NIPT with women.

**Electronic supplementary material:**

The online version of this article (doi:10.1186/s12884-017-1315-7) contains supplementary material, which is available to authorized users.

## Background

All women booking for antenatal care in the UK National Health Service (NHS) are offered Down syndrome screening. The combined screening test (CST), which measures fetal ultrasound markers and maternal serum hormone levels is the current recommended screening test (UK National Screening Committee). The CST has a detection rate of 84–90% and a false positive rate of 2–3%. Women with a ‘screen positive’ CST result (≥1:150) are offered an invasive diagnostic test, either chorionic villus sampling (CVS) or amniocentesis which have a small risk of miscarriage [1, 2].

Non-invasive prenatal testing (NIPT) which analyses cell-free fetal DNA (cffDNA) in maternal blood is a highly accurate screening test for Down syndrome that has been available in the private sector since 2011 and is currently being evaluated for use within state-funded healthcare systems in several countries [3–8]. NIPT is a maternal blood test that can be used from 10 weeks gestation with a detection rate of >99% for Down syndrome [9]. NIPT has been shown to be a much more accurate screening test for Down syndrome and the other common aneuploidies (T18 and T13) than traditional screening tests such as CST [10]. NIPT is not, however, diagnostic and an invasive test is recommended to confirm a positive NIPT result [11]. Overall, the use of NIPT significantly reduces the need for invasive testing and the associated iatrogenic miscarriages [12, 13].

Development and implementation of effective health professional training resources are essential to ensure counselling supports parents to make informed choices about NIPT. In the UK, midwives are the key professionals involved in discussing Down syndrome screening with women. Concerns about knowledge gaps amongst health professionals offering NIPT have been raised; particularly about non-fetal medicine specialists without training on some of the more complex issues specific to NIPT such as the importance of fetal fraction, and reasons why NIPT is not considered diagnostic, such as confined placental mosaicism, co-twin demise and unintended maternal diagnosis [14]. In addition, a key ethical concern about NIPT is the potential for informed decision making to be undermined if NIPT is viewed as a routine test [15–17]. Existing concerns about low rates of informed choice with current Down syndrome screening [18–22], further highlights the need for health professional education to focus on how best to support informed choice for NIPT.

A small number of studies have looked at the views of health professionals regarding NIPT and the need for educational resources for health professionals that are accurate and effective has been highlighted [23–30]. Here we aimed to develop and evaluate training resources to support midwives and other health professionals offering NIPT for Down syndrome.

## Methods

### Study design

This study formed one arm of the RAPID (Reliable, Accurate Prenatal, non-Invasive Diagnosis) NIPT Evaluation Study which aimed to explore the benefits and costs of implementing NIPT into the NHS. A detailed protocol [5], primary outcomes [8], experiences of women [31] and an evaluation of informed choice [32] have been published. Here we report on a mixed methods study involving questionnaires at three time points and interviews with a subset of participants evaluating training resources on NIPT developed for midwives and other health professionals offering Down syndrome screening in the eight hospitals participating in the NIPT evaluation study.

### Development of the training resources

The training resources include: a lesson plan, PowerPoint presentation and written factsheet (available on request). The training resources were developed alongside the patient information for the NIPT evaluation study. The training resources content was initially based on:

- National Genetics Education Centre - NIPT for Down syndrome competencies for health professionals [33].
- UK National Screening Committee educational resources [34].
- Published guidelines regarding NIPT for aneuploidy [35–37].
- Service user research that explores views and opinions about NIPT for Down syndrome [38, 39].

The first draft of the training resource was circulated to an expert panel for comment. The panel included an expert in fetal medicine and NIPT; two midwives; a genetic counsellor with expertise in developing competences for health professionals; researchers from the NHS Genetics and Genomics Education Centre; a research genetic counsellor; and representatives from the parent organisations Antenatal Results and Choices and Genetic Alliance UK who have expertise in developing health professional education and training packages.

Four focus groups were then held with a total of 67 health professionals with expertise in antenatal care, who attended a RAPID progamme dissemination meeting. The focus group discussion included the content of the training slides; developments in NIPT; areas that training should address, and level of information health professionals would require. The focus groups were digitally recorded, transcribed verbatim and analysed using thematic analysis [40]. The training materials were revised based on feedback from the focus groups and then piloted at one NHS trust participating in the main NIPT evaluation study prior to roll-out to other units. Minor changes to wording were made after piloting.

The final training material (lesson plan, PowerPoint presentation and written factsheet) covered 3 topic areas; 1. Background to cffDNA and NIPT; 2. NIPT evaluation study care pathways and reporting of results; and 3. counselling issues, with commonly asked questions and answers.

### Sample and recruitment

Training was given at eight UK hospitals located in England and Scotland prior to commencing recruitment for the NIPT evaluation study. Midwives and other health professionals offering Down syndrome screening and testing were invited by their managers and the local research team to attend a training session lasting 40 min. Training sessions were led by a member of the NIPT evaluation study research team.

### Questionnaires

Data was collected using three separate questionnaires (Q1, Q2, Q3) to allow a multi-stage approach similar to that used in the evaluation of other health professional training packages for genetics [41]. Paper questionnaires were completed prior to the training session (Q1) and immediately following the training session (Q2). A link to an online post-training ‘follow-up’ questionnaire (Q3) was emailed one month after the participant attended the training session if they had consented by providing their email address in Q2. Those participants who did not respond were resent Q3 with a reminder.

The questionnaires were developed by KO, reviewed by the expert panel and then piloted for understanding at one hospital. Following piloting the wording of some demographic questions were amended. The questionnaires are provided in the Additional file 1.

The content of the final questionnaires included;

- Pre-training questionnaire (Q1): demographic information, current practice for discussing Down syndrome testing (six statements with a four point Likert scale; always, if time, if asked, never), a measure of confidence in discussing NIPT, perceived knowledge and actual knowledge and previous experience of discussing NIPT (free text).
- Post-training questionnaire (An evaluation of the teaching session) (Q2): Questions exploring learners’ perceptions of the session, including whether these were pitched at the right level and were relevant to their practice (four-point Likert scale); questions exploring which aspects of the sessions were most useful; what aspects of the training participants would choose to change, and suggestions for improvements.
- Follow-up questionnaire (Q3): mirrored the pre-course questionnaire with the addition of questions asking participants to reflect on their experiences of the teaching sessions and the value of the written factsheet.

### Interviews

All health professionals who offer Down syndrome screening or testing at the sites participating in the NIPT evaluation study were invited by email to take part in a semi-structured telephone interview. An interview guide was used that focused on experiences with being involved in the RAPID NIPT evaluation study and views on the training sessions and written materials. Recruitment for interviews ceased when saturation was reached. The interviews were digitally recorded, transcribed verbatim and anonymised. Pseudonyms were assigned to each participant.

### Data analysis

The quantitative questionnaire data were coded and entered into SPSS version 18 (IBM, Chicago, IL, USA). The consistency and reliability of the scales were assessed using Cronbach’s alpha for the Likert scales and Kuder-Richardson 20 Formula for the dichotomous questions. Scores were calculated where over 50% of scale items were present, a mean score was calculated and then multiplied by the number of items in the scale to create the total score. Descriptive analysis was conducted on single items as well as for the 4 point Likert scale questions to produce frequencies and percentages. Histograms were produced to assess distribution of data. Total scores were calculated for the self-perceived confidence scale, the self-perceived knowledge scale, and the actual knowledge scale. Data was found not to be normally distributed therefore non-parametric tests were chosen for further analysis. A Wilcoxon-signed rank test was used to compare change in scores at the different time points. Significance was identified at the p level of <0.05. A Kruskal-Wallis test was used to compare differences in score between the different clinical areas that the midwives worked in and post hoc tests were performed using pairwise comparisons with adjusted p-values. The open-ended questions were analysed by copying the free text verbatim into an excel database, these were read and similar comments grouped together. Frequencies and percentages of the comments were calculated. Qualitative data from the focus groups and interviews were analysed using thematic analysis [40] facilitated by NVivo version 10 software (QSR International, Pty Ltd).

## Results

### Demographics

In total, 381 training attendees completed Q1 (97% response rate), 374 completed Q2 (95% response rate) and 151 completed Q3 (39% response rate). Thirty-five interviews were conducted. Participant characteristics are provided in Table 1.

**Table 1:** Participant demographics

	Pre/post training (*n =* 381)	Follow-up (*n* = 151)	Interviews (*n* = 35)
Hospital and region (no. trained)			
University College London Hospital, Central London (72)	70 (18.4%)	37 (24.5%)	8 (22.9%)
Queens Hospital Romford, Essex (64)	62 (16.3%)	22 (14.6%)	2 (5.7%)
St Georges Hospital, South West London (25)	24 (6.3%)	20 (13.2%)	7 (20.0%)
Princess Alexandra Hospital, Hampshire (131)	128 (33.6%)	45 (29.8%)	5 (14.3%)
Salisbury District Hospital, Wiltshire (20)	18 (4.7%)	7 (4.6%)	4 (11.4%)
Tayside Hospital, Scotland (9)	8 (2.1%)	4 (2.6%)	3 (8.6%)
Queen Charlottes and Chelsea Hospital, West London (38)	38 (10.0%)	8 (5.3%)	4 (11.4%)
Whittington Hospital, North London (33)	33 (8.7%)	8 (5.3%)	2 (5.7%)
Type of hospital			
District General Hospital	241 (63.3%)	82 (54.3%)	13 (37.1%)
Teaching Hospital	140 (36.7%)	69 (45.7%)	22 (62.9%)
Age group in years			
< 25	48 (13.1%)	17 (11.6%)	0
25 – 35	102 (27.8%)	49 (33.6%)	6 (20.0%)
36 – 45	77 (21.0%)	27 (18.5%)	8 (26.7%)
46 – 55	115 (31.4%)	46 (31.5%)	15 (50.0%)
> 56	24 (6.6%)	7 (4.8%)	1 (3.3%)
Gender			
Female	366 (97.3%)	142 (96.6%)	31 (88.6%)
Male	10 (2.7%)	5 (3.4%)	4 (11.4%)
Profession			
Midwife	355 (96.2%)	141 (96.6%)	28 (82.4%)
Other^b^	14 (3.8%)	5 (3.4%)	6 (17.6%)
Which area do you currently work in?			
Antenatal clinic	38 (10.3%)	14 (9.6%)	4 (11.8%)
Fetal medicine	25 (6.8%)	18 (12.3%)	2 (5.9%)
Community	110 (29.8%)	44 (20.1%)	4 (11.8%)
Rotational midwife^a^	15 (4.1%)	6 (4.1%)	0
Delivery and birth centre	8 (2.2%)	4 (2.7%)	0
Antenatal screening midwife/coordinator	3 (0.8%)	3 (2.1%)	5 (14.7%)
Postnatal ward	3 (0.8%)	2 (1.4%)	0
Student midwife	22 (6.0%)	8 (5.5%)	0
General inc. antenatal/community	118 (32.0%)	37 (24.3%)	0
Specialist midwife	13 (3.5%)	5 (3.4%)	13 (38.2%)
Other^b^	14 (3.8%)	5 (3.4%)	6 (17.6%)
Length of time in post in years			
< 1	32 (9.0%)	18 (12.5%)	9 (30.0%)
2 – 5	201 (56.3%)	83 (57.6%)	13 (43.3%)
6 – 10	64 (17.9%)	23 (16.0%)	6 (20.0%)
> 10	60 (16.8%)	20 (13.9%)	2 (6.7%)
Run booking clinics			
Yes	281 (75.7%)	116 (78.4%)	9 (25.7%)
No	90 (24.3%)	32 (21.6%)	26 (74.3%)

Missing data: between 2% and 6.3% of respondents did not answer one or more of the demographic questions

^a^Midwives who rotate between the maternity areas

^b^Obstetricians, Genetics, Day Assessment Unit, Education, Ultrasound

### Discussing Down syndrome screening prior to the training session

Table 2 outlines topics discussed in booking clinics regarding Down syndrome screening prior to the training session. The vast majority of the midwives always discuss what Down syndrome screening and diagnostic tests are available to women (95.8%); that screening is the woman’s choice (92.9%) and options for women with a high risk screening result (90.1%). It is less common for midwives to discuss the genetic cause of Down syndrome (34.7%), how the condition affects individuals (31.1%) and the accuracy of the current screening test (66.2%). Prior to training 50% felt confident or very confident discussing the genetic cause of Down syndrome with women.

**Table 2:** Current practice in discussing Down syndrome screening at booking

Information	Always	If asked	If I have time	Never
Genetic cause of Down syndrome	107 (34.7%)	158 (51.3%)	8 (2.6%)	35 (11.4%)
Common problems experienced by people with Down syndrome	94 (31.1%)	165 (54.6%)	15 (5.0%)	28 (9.3%)
What tests are available for Down syndrome	298 (95.8%)	12 (3.9%)	1 (0.3%)	0
Down syndrome screening is an optional test	290 (92.9%)	22 (7.1%)	0	0
The accuracy of the Combined Test	204 (66.2%)	79 (25.6%)	6 (1.9%)	19 (6.2%)
What the options are after a high risk screening result	283 (90.1%)	31 (9.9%)	0	0

Missing data: between 17.6% – 20.7% of respondents did not answer one or more of the questions

### Experiences of NIPT prior to the training session

Over half the midwives attending the training session had no previous experience of discussing NIPT for Down syndrome with women (*n =* 234, 64.1%); around a third had some experience (*n* = 115, 31.5%) and 16 (4.4%) had a lot of experience. Forty-seven respondents elaborated on their previous experience, which included women asking about NIPT for Down syndrome (*n* = 12), discussing option of NIPT in private practice with women who have had high risk Down syndrome screening results (*n* = 12), women who had already accessed NIPT in the private sector (*n* = 9) and offering NIPT through private practice being discussed routinely as a screening option during clinic (*n* = 9).

### Confidence

Following the training session analysis of Q2 showed 354 (96%) of attendees felt that they would feel confident in discussing NIPT with women in their clinic. There was a statistically significant improvement in the midwives confidence scores between Q1 (Mdn = 7, IQR = 9) and Q3 (Mdn = 23, IQR = 3), Z = 10.027, *p* < 0.001 (Table 3 and Additional file 2: Table 1). At Q3 135 participants felt more confident, 8 felt less confident and 6 participants’ confidence had not changed from Q1. At this stage 82% of the midwives felt confident or very confident.

**Table 3:** Summary of scales included in the questionnaire to measure confidence and knowledge

Measure	Description	Items	Reliability^a^	Range	Q1 Mdn (IQR)	Q3 Mdn (IQR)	Outcome
Self-perceived confidence scale	Clinical scenario given. Confidence felt in discussing features of NIPT.	13 items, 4 point Likert scales	0.95	0 – 39	7 (9)	23 (3)	Statistically significant improvement in self-perceived confidence *p* < 0.001
Self-perceived knowledge scale	Self-perceived knowledge regarding features of NIPT	6 items, 4 point Likert scales	0.92	0 – 18	1 (3)	8 (4)	Statistically significant improvement in self-perceived knowledge *p* < 0.001
Knowledge Test	Knowledge of cffDNA, features of NIPT, care pathway for NIPT	8 items, True, false, unsure	0.81	0 – 8	2 (4)	6 (2)	Statistically significant improvement in knowledge test *p* < 0.001

^a^Reliability was assessed using Cronbach’s alpha for Likert scales and Kuder-Richardson 20 Formula for dichotomous questions

There was a statistically significant difference in confidence score mean ranks between clinical work areas, *X* ^2^(9) = 41.028, *p* = <0.001. Pre-training fetal medicine midwives had higher confidence scores (273.21) compared to the following clinical work areas: student midwives (122.82; *p* <0.001); community midwives (169.64, *p* = 0.001); general midwives (171.72; *p =* 0.001); antenatal clinic midwives (183.43, *p =* 0.047). The post-training follow-up also showed a statistically significant difference between the clinical work areas confidence scores, (*X* ^2^(9) = 32.858, *p* < 0.001). This difference was between fetal medicine midwives (99.56) and community midwives (60.93; *p =* 0.49).

### Self-perceived knowledge

There was a statistically significant improvement in perceived knowledge about NIPT pre-training (Mdn = 1, IQR = 3) and post-training follow-up (Mdn = 8, IQR = 4, Z = 9.765, *p* < 0.001) (Table 3 and Additional file 2: Table 1). One hundred and thirty-one participants perceived themselves to be more knowledgeable about NIPT one month post-training, 4 perceived themselves to be less knowledgeable and 5 felt their knowledge had not changed.

At Q1 there was a statistically significant difference between the self-perceived knowledge score mean ranks between clinical work areas, (*X* ^2^(9) = 38.020, *p* < 0.001). Post hoc analysis revealed statistically significant difference in self-perceived knowledge scores between fetal medicine midwives (272.23) and the following areas: student midwives (144.27) (*p* < 0.001); community midwives (156.22) (*p* < 0.001); general midwives (178.75) (*p =* 0.001). At the post-training follow-up there was no statistically significant difference between the clinical work areas.

### Actual knowledge

There was a statistically significant difference in actual knowledge between Q1 (Mdn = 2, IQR = 4) and Q3 (Mdn = 6, IQR = 2), Z = 9.142, *p* < 0.001 (Tables 3); 115 participants had higher knowledge scores one month post training, 8 participants had lower scores and 13 participants scored the same pre- and post-training. Responses to the individual knowledge test questions are presented in Table 4. There were four knowledge areas where fewer than 65% of people indicated the correct response following training; test turn-around time; false positive rates; cell free DNA originates from the placenta; and cell free DNA concentration increases with gestation. There was a statistically significant difference in the knowledge test scores between the clinical work areas, *X* ^2^(9) = 38.020, *p =* <0.001. The difference lay between fetal medicine midwives (266.62) and the following work areas: student midwives (124.86) (*p* < 0.001); general midwives (166.13) (*p* < 0.001); community midwives (169.57) (*p =* 0.001). At the post-training follow-up there was no statistical difference in knowledge test score between the clinical work areas, *X* ^2^(9) = 11.230, p0.260.

**Table 4:** Knowledge test - respondents were asked to choose if each statement was true, false or if they were unsure

Knowledge test statement	Correct answer	Pre-training results		1-month follow-up after training results	
		Correct	Incorrect/unsure	Correct	Incorrect/unsure
Cell free fetal DNA originates from cells in the placenta	True	37 (25.3%)	109 (74.7%)	88 (63.8%)	50 (36.2%)
The concentration of cell free fetal DNA decreases with gestation	False	23 (15.6%)	124 (84.4%)	88 (42%)	80 (58%)
NIPT measures chromosome levels to identify elevated levels of chromosome 21, 18 and 13	True	57 (38.5%)	91 (61.5%)	113 (81.9%)	25 (18.1%)
NIPT for Down’s syndrome is over 99% accurate	True	72 (48.6%)	76 (51.4%)	98 (71%)	40 (29%)
The false positive rate for NIPT for Down’s syndrome is 10%	False	28 (19%)	119 (81%)	88 (63.8%)	50 (36.2%)
The turnaround time for the NIPT test is three weeks	False	25 (16.9%)	123 (83.1%)	54 (39.4%)	83 (60.6%)
NIPT for Down’s syndrome is being offered on a research basis to everyone with a screening test risk higher than 1:1000	True	52 (35.1%)	96 (64.9%)	109 (79%)	29 (21%)
There are three possible NIPT results: positive, negative or inconclusive	True	69 (46.6%)	79 (53.4%)	126 (91.3%)	12 (8.7%)
Women who receive a positive NIPT result will be offered an invasive test to confirm the result	True	65 (43.9%)	83 (56.1%)	129 (93.5%)	9 (6.5%)

### Training session format

There was an overwhelming consensus amongst the midwives (*n* = 363, 95.3%) that the information given in the training session would help them discuss NIPT with women. The level of information provided was thought to be suitable for both inexperienced and experienced midwives (Additional file 2: Table 2). The written information sheet was considered an essential part of the training (*n* = 130, 96.3%) as it was pitched at the right level (*n* = 128, 95.5%) to help midwives understand NIPT (*n* = 128, 95.5%), and was useful in their clinical practice (*n* = 117, 86.6%) (Additional file 2: Table 3). The qualitative work also found that midwives liked to have this as a source of reference, although some felt it was too detailed.

The vast majority of the midwives (*n* = 357, 94%) either agreed or strongly agreed that face-to-face training is essential to teach health professionals about NIPT (Additional file 2: Table 2) and the interview findings confirmed that the group format was valued as it allowed discussion. The majority of respondents (*n* = 287, 82%) also felt that an e-learning module would be a good way to educate health professionals about NIPT, with only 18% (*n* = 63) of respondents stating they did not think it would be useful. The midwives that elaborated further (*n* = 177) stated they preferred face-to-face learning as it offers the opportunity for questions and discussion (*n* = 46, 26%). However, it was acknowledged that e-learning may be beneficial as an adjunct to face-to-face learning (*n* = 22, 17%) as it offers several benefits, such as, easy accessibility and allows work to be done at own pace (*n* = 30, 17%); it is useful for referring to and refreshing knowledge (*n* = 39, 22%) and can reflect any changes as NIPT is further developed and introduced (*n* = 20, 11%).

### Interviews

The majority of comments about the training session were positive.

*“Well the content, I learnt a lot from it because I had no idea about this before I started so I learnt a lot from it and it was very clear and easy to understand and informative.”* HCP-LS21

Participants were happy to learn in a group setting, thought the information was pitched at the right level and valued the opportunity to ask questions (Table 5, quote 1 & 2). The written information sheet was thought to be an essential resource to return to. There were several positive comments that the written information was clear and easy to understand. However, there were some differences in opinions between participants about the level of information required (Table 5, quote 3 & 4).

**Table 5:** Quotes from interview participants

Quote number	Participant	Quote
1	HCP-CP33	*“I enjoyed the face to face when you come up yourself, because I felt that that was better, you can ask the questions and actually talk about it.”*
2	HCP-LG5	*“So I think it’s quite clear to understand. You don’t need to be very scientific to understand it, so quite user friendly. It’s not too much information in one go. It keeps to the points that you need to know as a Midwife explaining it and it’s easy to sort of reconstruct it into everyday language that you would then speak to the woman about.”*
3	HCP-KC2	“*[the written information sheet is] very self-explanatory, really nicely laid out, and if you’re going to read a, sort of, a proper information sheet on some research then, I don’t think it should be in point format, not like for the patients, it should be as detailed as it is…”*
4	HCP-JB9	“*the health professional information sheet that goes along with NIPT is very heavy.”*
5	HCP-HH35	*“it's sort of a drip, drip, drip, so it becomes really established behaviour, and it becomes, you know, like booking bloods, as part of booking, you offer booking bloods, and it becomes part of their really embedded routine when it comes to booking. I think you do that by just education, education, you know, mandatory training, at drop-in sessions … that kind of thing.”*
6	HCP-RM10	*“[Including NIPT in pre-registration midwifery training would mean] the next generation of midwives coming through already trained and ready to go and they just need to be sort of updated.”*
7	HCP-MO12	*“I think there could be a basic online package but I am not sure that people really take an awful lot in from online training because you just click buttons and you don’t always take everything in.”*
8	HCP-DT11	*“In person was much better for them because they could ask questions as we went along. I think it just stimulated them more to see a person standing there, coming out to them, rather than just online… I think it's just a bit drier. So I think, ideally, it would always be in person, but probably with online as a good second alternative for those who can't attend the actual training session.”*

In response to questions regarding further training, it was suggested that NIPT training be incorporated into National Screening Committee resources to ensure that standardised information is provided at a national level.

*“I think it's important to use really sort of nationally recognised resources, so something that comes from the National Screening Committee. A lot of these things that we're asking midwives to consent are difficult concepts to understand, and also you can sort of quite easily introduce personal bias, or slightly misunderstand something. So, I think it's making sure everyone's given the same level of information.”* HCP-HH35

To provide training on a wider scale it was suggested that the information should be: cascaded down through antenatal Screening Coordinators; added into mandatory training sessions and knowledge should be maintained with regular updates (Table 5, quote 5). It was also thought important that a wider group of health professionals be educated about NIPT, including GP’s and midwives who don’t work in antenatal clinic. One participant highlighted the benefit of incorporating NIPT education into pre-registration midwifery syllabus (Table 5, quote 6).

There were mixed opinions about online NIPT training, however, the consensus was that it would be useful to have the information online to refer to and work through at their own pace. There was concern about whether health professionals would complete online training; how easy it is to absorb information in online training and that there is no opportunity to ask questions. Overall, the majority thought it should only be used as an adjunct to face-to-face training (Table 5, quote 7 & 8).

## Discussion

NIPT for Down syndrome has been available in the private sector in the UK since late 2012 and has been evaluated for implementation into the NHS [5–8]. Comprehensive training of health professionals who will provide pre- and post-test counselling is essential for best practice in supporting pregnant women in decision making about NIPT. We developed a set of training resources for health professionals working in maternity units where NIPT was being offered to women as part of a research study evaluating the use of NIPT in the NHS [5, 8, 31, 32]. The face-to-face training and written factsheet that we provided to midwives was found to positively impact on their confidence in discussing NIPT with women in their clinic, and improved both their perceived and actual knowledge and understanding of NIPT. Nevertheless, our results suggest that midwives from some work areas (e.g. community midwives) might need additional training or support to optimise their confidence in discussing NIPT with women.

NIPT is a highly accurate screening test and comparison with traditional screening demonstrates that NIPT has higher detection rates, and lower false positive rates [10]. Ensuring women are informed about the difference in accuracy rates between the available screening tests is important so they are clear about the benefits and limitations of each testing option in order that they can made informed, individualised decisions. Notably, we found that the accuracy of Down syndrome screening tests is not always being discussed in current practice. At the follow-up to the training 94% of the midwives felt fairly or highly confident about discussing the accuracy of NIPT with women.

At the time of the follow-up questionnaire there was a significant improvement in the overall knowledge test score. However, analysis of the individual questions showed that at the post-training follow-up there were certain knowledge items that were still very poorly understood. Namely that cffDNA levels increase with gestation, the turnaround time for NIPT results, that cffDNA is placental in origin and the NIPT false positive rate. Ensuring that midwives are clear about the accuracy rate, including the risk of a false positive result is essential so that women are able to clearly interpret the meaning of the test results; including that NIPT is not a diagnostic test. This last point is particularly important as research in the USA with genetic counsellors highlighted that 74.8% of respondents reported that their patients consider NIPT results to be diagnostic [27]. In addition, two recent surveys of how NIPT is being used in the US found that 13% of fetal medicine specialists [26] and 15% of obstetric providers [29] view NIPT as a diagnostic test. It will also be important that women are aware of the current turnaround time for the test results (which in the RAPID study was 7–10 working days) as this may impact on decisions around whether to have NIPT or invasive testing for very high risk women, or those with ultrasound abnormalities. Consideration should also be given to knowledge gaps identified in pregnant women, such as the meaning of a negative NIPT result [42]. Future training should emphasise these areas and the knowledge could be reinforced through access to e-learning and/or further mandatory training.

Whilst a significant number of midwives supported e-learning, there was strong agreement both in the questionnaires and interviews that a face-to-face training session would be the superior method of information dissemination because of the opportunity to discuss and ask questions. This echoes findings from a literature review on web-based learning for dental, medical and nursing education that found that e-learning was not seen as an adequate replacement to traditional face-to-face learning [43]. Online resources have been identified as a good way to access up-to-date information [44, 45], which may be particularly relevant for NIPT as it is an area of rapid development. Future research in this area could include a direct comparison of the effectiveness of online versus face-to-face training. Whether training of health professionals on NIPT is conducted through face-to-face learning, e-learning or through blended learning which encompasses elements of both, it is likely that training on NIPT will need to be incorporated into the mandatory training offered by individual hospital’s antenatal Screening Coordinators. To provide training on a wider scale a cascade approach could be taken; resources can be shared with the National Screening Committee, who set education standards for Down syndrome screening, and antenatal Screening Coordinators who train midwives through mandatory training and study days at individual trusts. In addition to this the training could be incorporated into e-learning modules to enable midwives to refresh knowledge and keep abreast of changes in the care pathway if NIPT as introduced into the NHS.

In 2016 the National Screening Committee recommended that NIPT be implemented as a contingent test and an evaluative roll out of NIPT has ministerial approval to begin in England in 2018. This evaluative roll out is an important opportunity to examine the approaches and materials for education used in clinical practice to ensure health professionals are effectively prepared to provide up-to-date information about NIPT and support parents to make informed decisions. Clear care pathways will need to be developed to deliver NIPT in the NHS. It will be important to highlight which health professionals will offer NIPT, provide results and offer patient support. Training needs to be specific to the health professionals taking on each of these roles and strategies must be in place to ensure that skills and knowledge are maintained. The training resources described here could be adapted to support those midwives only providing information about NIPT with more detailed information available for those who will be offering the test and discussing results. This approach with two tiers of information may be helpful to cater for those wanting basic information and others who require more detailed information, as reflected in our data which showed that some midwives were happy with the level of information given while others felt it was too much.

### Limitations

A key limitation of the study was the loss of 60% of participants to follow-up. The sample completing Q3 was a small, self-selecting group; this limits generalizability and could create bias. Additionally, the RAPID NIPT evaluation study did not commence immediately after training in all the hospitals therefore not all the Q3 participants had the added benefit of experience in offering NIPT in clinical practice which could reinforce knowledge and build confidence. However, all interviews were conducted while the study was running so that feedback incorporates clinical experience. As there was not a validated questionnaire for evaluating NIPT for Down syndrome training a new set of questionnaires were designed for this study. The questionnaires have not been validated and cannot be used outside the context of this research. The scales used to produce scores were tested for internal consistency and reliability. In addition, the interviews served as a means of checking the questionnaire data and overall the two data sets correspond.

## Conclusions

A lesson plan, PowerPoint presentation and written factsheet on NIPT have been developed and evaluated, these have been updated and strengthened based on feedback. Training resulted in positive changes in knowledge, attitudes and confidence amongst midwives. These resources can be used as a starting point to update midwives and support the introduction of NIPT as it is implemented into the NHS and other settings. Updates are required for health professionals to ensure continued understanding/knowledge of Down syndrome screening. Training could be strengthened by providing greater focus on the areas where knowledge remained low, such as timing of results and false positive rates, and by providing two different levels of information. We identified that NIPT being available in the private sector is already having an impact on practice within the NHS, with a third of the midwives participating in this NHS-based study already having some experience of discussing NIPT with women in their clinics. This demonstrates that while NIPT has not yet been implemented within the NHS, it is important that education strategies are undertaken as soon as possible.

## Additional files

Additional file 1: Study questionnaires . The three questionnaires (Q1, Q2, Q3) used in the study are provided in full. (DOCX 83 kb)

Additional file 2: Table 1. Confidence and knowledge scores. **Table 2** Opinions about the face-to-face presentation from participants immediately after the training session. **Table 3** Opinions on the written fact-sheet 20. (DOCX 18 kb)

## Abbreviations

cffDNA: Cell-free fetal DNA
CST: Combined screening test
CVS: Chorionic villus sampling
NHS: National Health Service
NIPT: Non-invasive prenatal testing
NRES: National Research Ethics Service
RAPID: Reliable, Accurate Prenatal, non-Invasive Diagnosis
