# Supplementary material for: Development and evaluation of training resources to prepare health professionals for counselling pregnant women about non-invasive prenatal testing for Down syndrome: a mixed methods study
Source: BMC Pregnancy Childbirth. 2017 Apr 27;17:132. doi: 10.1186/s12884-017-1315-7 (PMC5408404; doi:10.1186/s12884-017-1315-7)
Supplement: Supplementary file 1 — Study questionnaires . The three questionnaires (Q1, Q2, Q3) used in the study are provided in full. (DOCX 83 kb) [file 12884_2017_1315_MOESM1_ESM.docx]

**Supplementary material – study questionnaires**


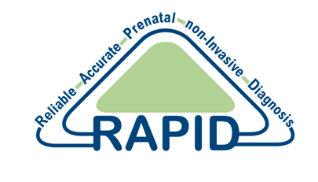


**Non Invasive Prenatal Testing (NIPT) for Down’s syndrome**

**Pre-course questionnaire**

| This short questionnaire should take no more than 10 minutes to complete. It aims to find out:   - A little bit about yourself and your clinical experience - Your current level of confidence in offering NIPT for Down’s syndrome - How you rate your knowledge of NIPT for Down’s syndrome   The results will enable us to evaluate the NIPT education sessions so we can develop them further to meet the needs of health professionals who will be offering the test in the future.  Your feedback is very important to us. |
| --- |

| **Section A: About you** |
| --- |

1. **Are you:**  **Male**  **Female**
2. **Age:**  < 25  25-30  31-35  36-40

41-45  46-50  51-55  56-60  > 60

1. **How many years have you been in this post? _________**
2. **What is your main area of work? *Please tick all that apply.***

Antenatal clinic  Fetal medicine  Community

Rotational midwife  Delivery suite  postnatal ward

Other *Please state:* __________

1. **Do you run booking clinics?**

Yes  No

**If yes, what information about Down’s syndrome screening do you normally discuss with women in your booking clinics?**

|  | Always | If asked | If I have time | Never |
| --- | --- | --- | --- | --- |
| What is the cause of Down’s syndrome | □ | □ | □ | □ |
| What the common problems people with Down’s syndrome can have | □ | □ | □ | □ |
| What tests are available for Down’ Syndrome | □ | □ | □ | □ |
| The option to opt out of Down’s syndrome testing | □ | □ | □ | □ |
| The accuracy of the Combined screening test | □ | □ | □ | □ |
| What the options are if they receive a high risk screening result | □ | □ | □ | □ |

1. **Have you had any experience discussing NIPT for Down’s syndrome?**

None at all  A little  A lot

*Please tell us about your experience:*  __________________________________________________________________________________________________________________________________________________________________________

1. **Have you had any experience discussing NIPT for other uses such as fetal rhesus genotyping or for fetal sex determination?**

*Please tell us about your experience:*  __________________________________________________________________________________________________________________________________________________________________________

| **Section B: Clinical scenario** |
| --- |

The following clinical scenario aims to find out how confident you feel about discussing and offering NIPT for Down’s syndrome. Don’t worry if you have never done this before, just read the scenario and then work through the questions.

1. **Cara is 10 weeks gestation and attends her booking appointment with you. You discuss screening for Down’s syndrome and Cara she asks you about a new test she has heard about called Non-invasive prenatal testing (NIPT). She asks if the test is available to her. Thinking about this scenario please indicate how confident you feel about the following activities.**

|  | Not at all confident | Fairly confident | Confident | Very confident |
| --- | --- | --- | --- | --- |
| Explaining what causes Down’s syndrome | □ | □ | □ | □ |
| Explaining Cara’s current Down’s syndrome testing options (screening / invasive testing) | □ | □ | □ | □ |
| Advising Cara whether or not she can have NIPT for Down’s syndrome | □ | □ | □ | □ |
| Explaining what cell free fetal DNA is and how NIPT for Down’s syndrome works | □ | □ | □ | □ |
| Explaining the accuracy and reliability of NIPT for Down’s syndrome | □ | □ | □ | □ |
| Explaining NIPT test results and describing the options for Cara if she has a positive result | □ | □ | □ | □ |
| Explaining NIPT test results and describing the options for Cara if she has a negative result | □ | □ | □ | □ |
| Explaining an inconclusive result and describing the possible next steps Cara can take | □ | □ | □ | □ |

| **Section C: Your knowledge** |
| --- |

The following questions aim to find out your current knowledge of NIPT and how confident you feel about your knowledge. Don’t worry if you are unsure about many of these questions, they will be addressed in the training.

1. **How would you rate your knowledge about NIPT for Down’s syndrome?**

|  | I know nothing about this | I know a little about this | I know a lot about this | I know enough to teach this |
| --- | --- | --- | --- | --- |
| The scientific principles of cell free fetal DNA | □ | □ | □ | □ |
| What non-invasive tests based on cell free fetal DNA are currently available | □ | □ | □ | □ |
| How the laboratory test for NIPT for Down’s syndrome works | □ | □ | □ | □ |
| The features of the test such as accuracy and turnaround time | □ | □ | □ | □ |
| Criteria women must meet to be recruited to the NIPT implementation study | □ | □ | □ | □ |
| How NIPT will fit into the current Down’s syndrome testing pathway | □ | □ | □ | □ |

1. **Cara has a combined screening test, her result is 1:850, she has been recruited to the NIPT implementation study and wants to ask you some questions. Please state whether the following statements are true or false:**

|  | True | False | Unsure |
| --- | --- | --- | --- |
| Cell free fetal DNA originates from cells in the placenta | □ | □ | □ |
| The concentration of cell free fetal DNA decreases with gestation | □ | □ | □ |
| NIPT measures chromosome levels to identify elevated levels of chromosome 21, 18 and 13 | □ | □ | □ |
| NIPT for Down’s syndrome is over 99% accurate | □ | □ | □ |
| The false positive rate for NIPT for Down’s syndrome is 10% | □ | □ | □ |
| The turnaround time for the NIPT test is three weeks | □ | □ | □ |
| NIPT for Down’s syndrome is being offered on a research basis to everyone with a screening test risk higher than 1:1000 | □ | □ | □ |
| There are three possible NIPT results: positive, negative or inconclusive | □ | □ | □ |
| Women who receive a positive NIPT result will be offered an invasive test to confirm the result | □ | □ | □ |

**Thank you for your help with this research.**

**
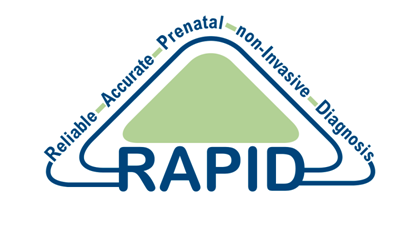
Non Invasive Prenatal Testing (NIPT) for Down’s syndrome end of training session questionnaire**

| This questionnaire should take no more than 5 minutes of your time to complete.  As part of this evaluation we would like to gauge your opinion of the “NIPT for Down’s syndrome” education session that you have just had. Your feedback will enable us to make improvements to the structure and content of the session, hopefully improving it for future learners. |
| --- |

| **Section A: Your opinions of the education session** |
| --- |

1. **Considering the presentation you have seen today, for each of the following statements please indicate the degree to which you agree/disagree with the listed statement.**

|  | Strongly disagree | Disagree | Agree | Strongly agree |
| --- | --- | --- | --- | --- |
| I think a face to face presentation is essential to teach health professionals  about NIPT | □ | □ | □ | □ |
| The presentation was pitched at the right level for me | □ | □ | □ | □ |
| The presentation was pitched at the right level for someone with less experience than me | □ | □ | □ | □ |
| The presentation was pitched at the right level for someone with more experience than me | □ | □ | □ | □ |
| The presentation has helped me understand NIPT for Down’s syndrome | □ | □ | □ | □ |
| The information given in the presentation will help me advise women on NIPT in my future practice | □ | □ | □ | □ |

1. **Will you feel confident discussing the NIPT implementation study with women in your future practice?**

Yes  No

*Please explain your response:* **________________________________________________________________________________________________________________________________________________________________________________________________________________________________________________**

1. **What aspects of this session were particularly useful to you?**

**______________________________________________________________________________________________________________________________________________________________________________________________________________________________________________________**

1. **What would you change about this session?**

**______________________________________________________________________________________________________________________________________________________________________________________________________________________________________________________**

1. **Do you think an e-learning module would be a good way to update your knowledge on NIPT and educate other health professionals about NIPT in the future?**

Yes  No

*Please explain your response:*  **________________________________________________________________________________________________________________________________________________________________________________________________________________________________________________**

1. **Do you have any suggestions for what we should incorporate when developing further health professional training on NIPT for Down’s syndrome?**

**________________________________________________________________________________________________________________________________________________________________________________________________________________________________________________**

**Thank you for your help with this research.**


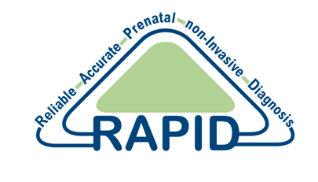


**Non Invasive Prenatal Testing (NIPT) for Down’s syndrome**

**Post-course questionnaire**

| This short questionnaire should take no more than 10 minutes to complete. It aims to find out:   - Your current level of confidence in offering NIPT for Down’s syndrome - How you rate your knowledge of NIPT for Down’s syndrome - Your opinions of the NIPT education session   The results will enable us to evaluate the NIPT education sessions so we can develop them further. The information from this evaluation will help us to develop educational materials so they specifically meet your needs. Your feedback is very important to us. |
| --- |

| **Section A: Clinical scenario** |
| --- |

The following clinical scenario aims to find out how confident you feel about discussing and offering NIPT for Down’s syndrome.

1. **Cara is 10 weeks gestation and attends her booking appointment with you. You discuss screening for Down’s syndrome and Cara she asks you about a new test she has heard about called Non-invasive prenatal testing (NIPT). She asks if the test is available to her. Thinking about this scenario please indicate how confident you feel about the following activities.**

|  | Not at all confident | Fairly confident | Confident | Very confident |
| --- | --- | --- | --- | --- |
| Explaining what causes Down’s syndrome | □ | □ | □ | □ |
| Explaining Cara’s current Down’s syndrome testing options (screening / invasive testing) | □ | □ | □ | □ |
| Advising Cara whether or not she can have NIPT for Down’s syndrome | □ | □ | □ | □ |
| Explaining what cell free fetal DNA is and how NIPT for Down’s syndrome works | □ | □ | □ | □ |
| Explaining the accuracy and reliability of NIPT for Down’s syndrome | □ | □ | □ | □ |
| Explaining NIPT test results and describing the options for Cara if she has a positive result | □ | □ | □ | □ |
| Explaining NIPT test results and describing the options for Cara if she has a negative result | □ | □ | □ | □ |
| Explaining an inconclusive result and describing the possible next steps Cara can take | □ | □ | □ | □ |

| **Section B: Your knowledge** |
| --- |

1. **How would you rate your knowledge about NIPT for Down’s syndrome?**

|  | I know nothing about this | I know a little about this | I know a lot about this | I know enough to teach this |
| --- | --- | --- | --- | --- |
| The scientific principles of cell free fetal DNA | □ | □ | □ | □ |
| What non-invasive tests based on cell free fetal DNA are currently available | □ | □ | □ | □ |
| How the laboratory test for NIPT for Down’s syndrome works | □ | □ | □ | □ |
| The features of the test such as, accuracy and turnaround time | □ | □ | □ | □ |
| Which women will be recruited to the NIPT implementation study | □ | □ | □ | □ |
| How NIPT will fit into current Down’s syndrome testing pathway | □ | □ | □ | □ |

1. **Cara has a combined screening test, her result is 1:850, she has been recruited to the NIPT implementation study and wants to ask you some questions. Please state whether the following statements are true or false:**

|  | True | False | Unsure |
| --- | --- | --- | --- |
| Cell free fetal DNA originates from cells in the placenta | □ | □ | □ |
| The concentration of cell free fetal DNA decreases with gestation | □ | □ | □ |
| NIPT measures chromosome levels to identify elevated levels of chromosome 21, 18 and 13 | □ | □ | □ |
| NIPT for Down’s syndrome is over 99% accurate | □ | □ | □ |
| The false positive rate for NIPT for Down’s syndrome is 10% | □ | □ | □ |
| The turnaround time for the NIPT test is three weeks | □ | □ | □ |
| NIPT for Down’s syndrome is being offered on a research basis to everyone with a screening test risk higher than 1:1000 | □ | □ | □ |
| There are three possible NIPT results: positive, negative or inconclusive | □ | □ | □ |
| Women who receive a positive NIPT result will be offered an invasive test to confirm the result | □ | □ | □ |

**Section C: Your Opinions**

1. **You were provided with some written information about NIPT for Down’s syndrome at your previous education session, for each of the following statements please indicate the degree to which you agree/disagree with the listed statement.**

|  | Strongly disagree | Disagree | Agree | Strongly agree |
| --- | --- | --- | --- | --- |
| I think written information is essential to help me learn about NIPT | □ | □ | □ | □ |
| The written information was pitched at the right level for me | □ | □ | □ | □ |
| The written information was pitched at the right level for someone with less experience than me | □ | □ | □ | □ |
| The written information was pitched at the right level for someone with more experience than me | □ | □ | □ | □ |
| The written information has helped me understand NIPT for Down’s syndrome | □ | □ | □ | □ |
| Since the training session I have found the written information useful for my practice | □ | □ | □ | □ |

1. **Do you have any additional comments about the NIPT for Down’s syndrome education session that you have not had a chance to give?**

________________________________________________________________________________________________________________________________________________________________________________________________________________________________________________

________________________________________________________________________________________________________________________________________________________________

**Thank you for your help with this research.**
