# Supplementary material for: Development and evaluation of training resources to prepare health professionals for counselling pregnant women about non-invasive prenatal testing for Down syndrome: a mixed methods study
Source: BMC Pregnancy Childbirth. 2017 Apr 27;17:132. doi: 10.1186/s12884-017-1315-7 (PMC5408404; doi:10.1186/s12884-017-1315-7)
Supplement: Supplementary file 2 — Confidence and knowledge scores. Table 2 Opinions about the face-to-face presentation from participants immediately after the training session. Table 3 Opinions on the written fact-sheet 20. (DOCX 18 kb) [file 12884_2017_1315_MOESM2_ESM.docx]

**Supplementary material**

Table 1: Confidence and knowledge scores

|  | **Self-perceived Confidence Score** | | **Self-perceived Knowledge Score** | | **Knowledge Test Score** | |
| --- | --- | --- | --- | --- | --- | --- |
|  | **Pre-training** | **1-month follow-up** | **Pre-training** | **1-month follow-up** | **Pre-training** | **1-month follow-up** |
| Median | 7 | 23 | 1 | 8 | 2 | 6 |
| Interquartile Range | 9 | 3 | 3 | 4 | 4 | 2 |
| Z score | 10.027 | | 9.765 | | 9.142 | |
| p. value | <0.001 | | <0.001 | | 0.001 | |

Table 2: Opinions about the face-to-face presentation from participants immediately after the training session.

|  | **Strongly Disagree** | **Disagree** | **Agree** | **Strongly Agree** |
| --- | --- | --- | --- | --- |
| I think a face to face presentation is essential to teach health professionals about NIPT | 6 (1.6%) | 10 (2.6%) | 114 (29.9%) | 243 (63.8%) |
| The presentation was pitched at the right level for me | 7 (1.8%) | 2 (0.5%) | 139 (36.5%) | 225 (59.1%) |
| The presentation was pitched at the right level for someone with less experience than me | 12 (3.1%) | 32 (8.4%) | 181 (47.5%) | 133 (34.9%) |
| The presentation was pitched at the right level for someone with more experience than me | 8 (2.1%) | 32 (8.4%) | 172 (45.1%) | 146 (38.3%) |
| The presentation has helped me understand NIPT for Down’s syndrome | 5 (1.3%) | 5 (1.3%) | 136 (35.7%) | 228 (59.8%) |
| The information given in the presentation will help me advise women on NIPT in my future practice | 5 (1.3%) | 4 (1%) | 138 (36.2%) | 225 (59.1%) |

Table 3: Opinions on the written fact-sheet

|  | **Strongly Disagree** | **Disagree** | **Agree** | **Strongly Agree** |
| --- | --- | --- | --- | --- |
| I think written information is essential to help me learn about NIPT | 2 (1.5%) | 3 (2.2%) | 68 (50.4%) | 62 (45.9%) |
| The written information was pitched at the right level for me | 4 (3%) | 2 (1.5%) | 96 (71.6%) | 32 (23.9%) |
| The written information was pitched at the right level for someone with less experience than me | 3 (2.2%) | 39 (29.1%) | 79 (59%) | 13 (9.7%) |
| The written information was pitched at the right level for someone with more experience than me | 3 (2.3%) | 35 (26.3%) | 78 (58.6%) | 17 (12.8%) |
| The written information has helped me understand NIPT for Down’s syndrome | 3 (2.2%) | 3 (2.2%) | 97 (72.4%) | 31 (23.1%) |
| Since the training session I have found the written information useful for my practice | 3 (2.2%) | 15 (11.1%) | 89 (65.9%) | 28 (20.7%) |
